# The genetic integrity of bacterial species: the core genome and the accessory genome, two different stories

**DOI:** 10.3389/fcimb.2012.00116

**Published:** 2012-09-06

**Authors:** Bo Segerman

**Affiliations:** National Veterinary InstituteUppsala, Sweden

**Keywords:** accessory genome, bacterial species, core genome, lateral gene transfer, species border

## Abstract

Strains within a bacterial species typically have a set of conserved core genes and a variable set of accessory genes. The accessory genes often appear to move laterally between strains, thereby forming new trait combinations. Sometimes, genetic material also moves laterally between species, thereby resulting in diffuse borders between them. The growing number of genome sequences offers new possibilities to study these processes. Ten species for which abundant genomic data exists were here selected for analysis of the species border integrity. The average core genome similarities and relative core genome sizes (RCGSs) were determined for strain pairs within the species and for strain pairs crossing the species border. The variability within the species as well as the border integrity varies for different bacterial species. Some have very distinct borders while others are more or less indefinable. From the growing amount of genomic data, it becomes even clearer that the concept of bacterial species is, in many cases, far from absolute.

## Introduction

In higher eukaryotes, a species is often defined as a group of organisms that are so reproductively isolated that interbreeding with other species cannot occur or does not result in a fertile offspring. This is believed to maintain the genetic integrity of the species over time. A genetic pool that is much larger than that present in each individual is maintained within the species and sexual reproduction accounts for the formation of new allele combinations. Thus, the genetic material is inherited vertically from the combined genetic pool of the parents and the apparent universality of this inheritance mode led Darwin to propose that all organisms could be organized into a “tree of life.”

In prokaryotes, the situation is somewhat different. Their genetic material is asexually inherited from the ancestral cell. Accumulation of mutations during this clonal expansion can give rise to sub-populations with selective advantages. If prokaryotes were to rely on only this mechanism for adaptation, new trait combinations would require “reinventing the wheel” over and over. Thus, it is not surprising that lateral gene transfer (LGT) mechanisms exist in prokaryotes (Ochman et al., 2000). LGT allows advantageous genes to sweep through populations (Shapiro et al., 2012). As a consequence of the lateral movements of genetic material, the organisms becomes chimerical and a strict tree model cannot adequately represent their phylogenetic relationships (Bapteste et al., 2009). To reflect this, the phrase “rhizome of life” is sometimes used as an alternative to “tree of life” (Raoult, 2010). It is clear that different models to represent this complex evolutionary history are necessary, depending on what scientific questions we are addressing, i.e., we need to use “pattern pluralism” in our way of thinking (Doolittle and Bapteste, 2007). However, the relative extent to which the tree model should, or must, be discarded in favor of alternative models is still debated and varies depending on the species in question. A further consequence of LGT is that the actual concept of the bacterial species becomes partially undermined (Doolittle and Papke, 2006). A broader viewpoint for describing bacterial population structures constitutes the presence of sympatric species complexes with high plasticity and lateral gene exchange from which specialized allopatric species, such as pathogens, can escape (Georgiades and Raoult, 2010). Reductive evolution accompanying the pathogenic lifestyle will, in many cases, confine the species (Merhej et al., 2009).

**Figure 1:**
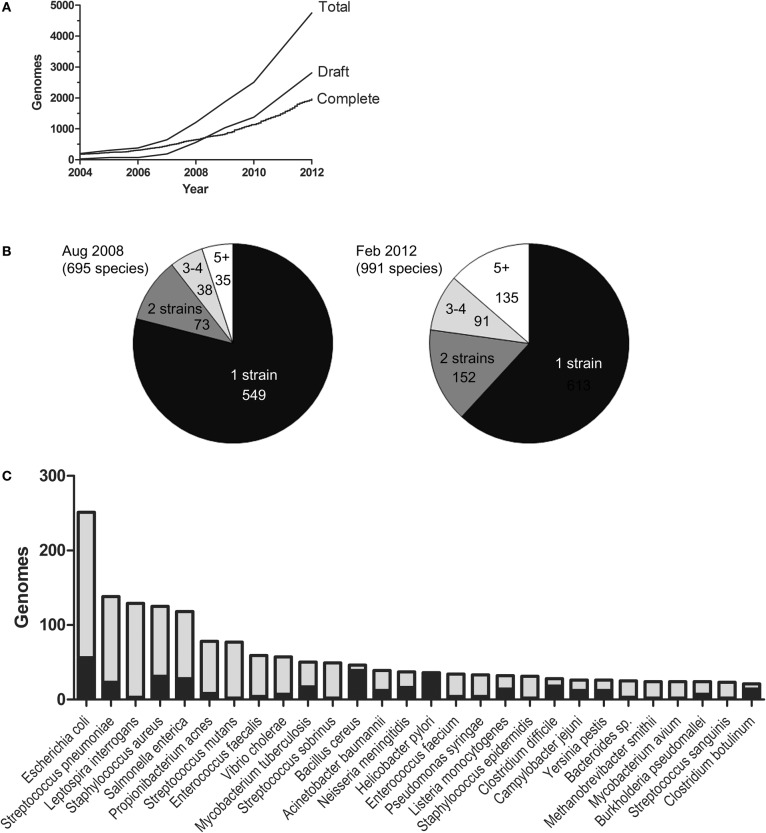
**The genome database**. The figure is based on the NCBI GenomeReport dated 21 Feb, 2012 and only includes genomes projects where completed sequences or draft assemblies were available for download. **(A)** The growth of the databases for draft and complete bacterial genomes. **(B)** The number of species with 1, 2, 3, 4, and 5 (or more) strains sequenced Aug 2008 and Feb. 2012, respectively. **(C)** The species with most genome sequences. Black represents completed genomes and grey draft genomes.

Recently, a clearer understanding of bacterial genome evolution has emerged. This is mainly because of the intense technological development of high-throughput sequencing (Metzker, 2010). Sequencing is now done in enormous amounts of randomly primed parallel sequencing reactions from the same sample. The technologies are often collected under the name Next Generation Sequencing (NGS). NGS has unquestionably had an enormous impact on the growth rate of the bacterial genome database (Figure 1A). As parallel sequencing machines are moving from the core facilities into regular laboratories, it is likely that the number of sequences will continue to increase exponentially. In the early days of bacterial genome sequencing, most projects typically aimed to produce a complete genome sequence as the final product. Making a complete genome sequence still requires a lot of resources. In contrast, draft sequences have become easy and affordable to produce and are often sufficient for answering many biological questions. Consequently, the draft genome database is growing more rapidly than the complete one. At the time of the analysis presented in this paper, there were over 2000 completed genomes and almost 3000 draft genomes in the form of contigs/scaffolds. There are also a large number of draft genome assemblies not yet submitted to the database.

How are the current sequencing efforts being directed? Figure 1B shows the number of species in the genome database in August 2008 and February 2012. The number of species has grown from approximately 700 to almost 1000 (a 1.4-fold increase) and the number of sequences from 1255 to ~4900 (a 3.9-fold increase). This illustrates that sequencing activities aimed at producing more sequences from strains belonging to already represented species are far more intense than sequencing projects directed towards new species. The ten most sequenced species account for 22% of the genome database. The bacterial species for which the highest number of genome sequences are available (at the time of this writing) are shown in Figure 1C. The large number of available genomic sequences has given us the opportunity to compare genomic variation within species as well as between them. However, sequencing efforts are undoubtedly biased towards strains of medical or economical importance and this may very well bias the conclusions we make.

The genomic sequencing efforts have made us realize that, similar to eukaryotes, bacterial species maintain a “genetic pool” much larger than the one present in each strain. Each strain has a conserved set of core genes and additionally, a number of accessory genes. The dynamics of the accessory genes can give rise to strains with “customized genomic repertoires” (Mathee et al., 2008). Thus, different strains may have different sets of accessory genes and the superset of all different genes present in a species is often referred to as the pan-genome (Tettelin et al., 2005). It is likely that the accessory genes constitute a reservoir for functionality that can be transferred laterally to create new trait combinations. However, a large fraction of the accessory genes have often no functional annotation; our knowledge about many of these genes is poor. The accessory genome has become an important field of study (Sim et al., 2008; Bennett et al., 2010; Kung et al., 2010).

In this study, a limited dataset of species representing different life strategies and having a high number of both draft and complete genome sequences was selected for a more detailed analysis (Table 1). The genomic information was here used to study the genetic integrity of these bacterial species in terms of core genome sequence variability and variations in the relative size of the core genome/accessory genome.

**Table 1:** **Species included in the analysis**.

**Genus**	**Species analyzed**	**Number of species represented in the genome sequence database**
*Staphylococcus*	*S. aureus*	12
*Streptococcus*	*S. pneumoniae*	37
*Escherichia*	*E. coli*	3
*Salmonella*	*S. enterica*	2
*Mycobacterium*	*M. tuberculosis*	17
*Neisseria*	*N. meningitidis*	15
*Helicobacter*	*H. pylori*	12
*Clostridium*	*C. botulinum*	14
*Bacillus*	*B. anthracis*	20
*Burkholderia*	*B. pseudomallei*	17

## Comparative analysis

The degree of sequence similarity within the core genome is considered to be one of the best phylogenomic measures for comparing microbial genomes (Rokas et al., 2003). By averaging comparisons of a large number of genes, the risk for disturbances in the result caused by laterally moved genetic material is minimized. In this study, pairwise average core genome similarity (ACGS) values and relative core genome size (RCGS) values were calculated using the Gegenees fragmented alignment method (Ågren et al., 2012). In brief, the genomes were fragmented into overlapping 200-basepair pieces and for each fragment a BLASTN score was calculated. The core genome was defined as the regions constituted by fragments with scores of at least 25% of the score value of a perfect match. The average similarity was normalized towards the value obtained when the genome was compared to itself. The RCGS value represents the core genome size relative to the whole genome size (core genome + accessory genome).

If genetic material were only inherited vertically, the differences in size of the accessory genetic material between two strains would depend on gene loss events and duplication events followed by acquisition of new functionality (neo-functionalization or sub-functionalization). It would then be expected that RCGS would gradually decrease as ACGS decrease. However, if lateral movement of accessory genetic material occur, a much greater variation in RCGS values would be expected because lateral movements would be more or less uncoupled with the vertical inheritance.

In this study, the relationship between ACGS and RCGS values and the integrity of the species border in terms of these values were examined by analysis of a large number of pairwise comparisons within a selected set of bacterial genera. Ten species, with good representation in the sequence database (Table 1), were selected. The ACGS and RCGS values were then calculated, pairwise, for every possible genome combination in the genus. Thus, there were both intra- and inter-species comparisons. The data were used to create a diagram with the highest ACGS (shortest vertical inheritance distance) first, and then the pairwise comparisons plotted in descending order. In the same diagram, the corresponding RCGS values were plotted and the part of the diagram that represented intra-species comparisons was indicated. Hereafter, this diagram type is referred to as a “species integrity diagram.” The software Gegenees (Ågren et al., 2012) can, from version 1.1.5, generate this type of diagram and also gives interactive annotations of individual data points.

## Species integrity of ten selected species

**Figure 2:**
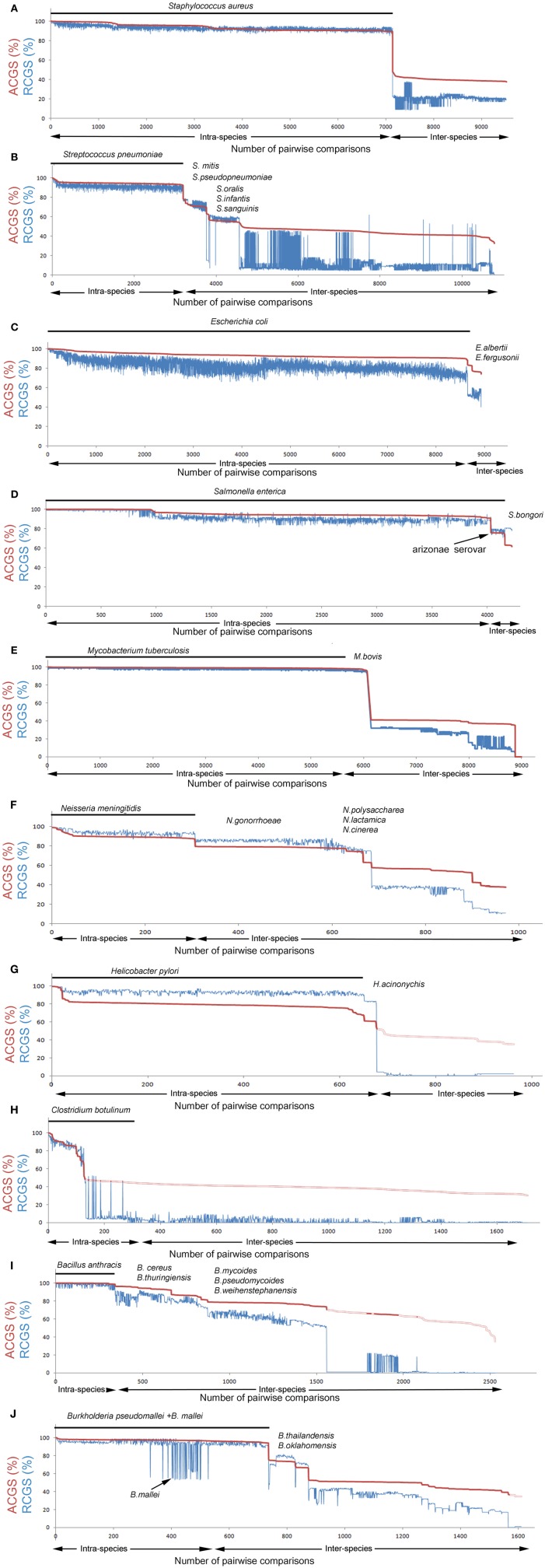
**Species integrity diagrams for 10 selected species**. Pairwise genome comparisons defining average core genome similarity (ACGS) are shown in red and relative core genome size (RCGS) in blue. ACGS measures phylogenomic distance while RCGS measures the size of the core genome relative to the total genome size (core + accessory genome). Intra-species comparisons are plotted first (indicated with the black line) and inter-species comparisons thereafter. Data are sorted by ASCG. **(A–J)** Intra-species comparisons of strains from the selected species [*Staphylococcus aureus, Streptococcus pneumoniae, Escherichia coli, Salmonella enterica, Mycobacterium tuberculosis, Neisseria meningitidis, Helicobacter pylori, Clostridium botulinum, Bacillus anthracis* (a part of the *Bacillus cereus* group), and *Burkholderia pseudomallei*] and inter-species comparison with strains from other species in the same genus.

In the intra-species pairwise comparison of *Staphylococcus aureus* strains, ACGS values varied between 90% and 100% (Figure 2A). The RCGS values fluctuated between 90–99% (i.e., up to 10% accessory genetic material) with modest correlation with the ACGS values. This indicates that the accessory genetic material is mobile between strains within the species border. Lateral transfer of genetic material in *S. aureus* has been described previously in relation to mobile genetic elements (Deurenberg and Stobberingh, 2008; Lindsay, 2010). The closest strain in the inter-species comparison had distinctly lower ACGS and RCGS values. Thus, on the basis of the genomic data available today, there seems to be a distinct border between the species *S. aureus* and its closest neighbors.

In *Streptococcus pneumoniae*, the intra-species comparison results were very similar to those found in *S. aureus* (Figure 2B). Lateral transfer within *S. pneumoniae* has been described previously (Coffey et al., 1991). Inter-species comparisons show that the distance between *S. pneumoniae* and its closest related strains outside the species border (belonging to *S. mitis*) is quite small and there is a continuous decline in similarity with not fully distinct plateaus. This indicates that *S. pneumoniae* is exchanging genes with other *Streptococcus* spp. The population structure and dynamics of *S. pneumoniae* in terms of the pan genome has been studied previously (Donati et al., 2010).

In the genus *Escherichia*, almost all sequences came from *E. coli*. The high amount of accessory genetic material and the large RCGS fluctuations between strain pairs with similar ACGS values (Figure 2C) suggest that there is an extraodinary mobility of accessory genetic material in *E. coli*. The border with other *Escherichia*. spp is not distinct although the sequencing database is underrepresented for non *E. coli* species. Like *Escherichia*, the related *Salmonella* genome database is dominated by a single species, *S. enterica*. *S. enterica* strains typically have an ACGS value of ~55% compared to *E. coli* strains. The diagram shows that *S. enterica strains* have a lower variability in accessory genome size compared to *E. coli* (Figure 2D).

In contrast to the examples above, *Mycobacterium tuberculosis* is very distinct (Figure 2E). Low variability was seen for both for RCGS and ACGS values as was a clear difference to related, non-*M. tuberculosis* strains (except for *M. bovis* as discussed below). This indicates lateral gene movements are less frequent in this species. However, another type of problem in species designation becomes apparent here. *M. bovis* is, on a genomic level, an indistinguishable part of *M. tuberculosis*. Detailed genome analysis of a *M. bovis* strain has also shown that there is a very high genomic similarity to *M. tuberculosis* (Garnier et al., 2003).

*Neisseria meningitidis* is distinct but closely related to strains outside the species (Figure 2F). The closest strains belonged to the species *N. gonorrhoeae*. Many *N. meningitidis* strains showed only 10% lower RCGS values when compared to *N. gonorrhoeae* strains than when compared to the other strains within the species. This suggests crossover of genetic material can occur over the species border; nevertheless the ACGS values show clear distinct plateaus. The accessory genome has been observed to evolve differently from the core genome in *Neisseria* (Bennett et al., 2010).

In *Helicobacter pylori*, the ACGS values were, in most intra-species comparisons, relatively low (Figure 2G). This suggests that *Helicobacter* has a comparatively high mutation rate. This has also been discussed previously (Wang et al., 1999). The RCGS values fluctuate, as described in many species above, indicating the lateral movement of genes.

*Clostridium botulinum* is a classical example of how long distance lateral gene movements can affect the integrity of what we call a bacterial species. The Botulinum Neuroxin (BoNT) gene, *bont*, has during several occasions jumped between quite distantly related *Clostridium* strains and this can be seen in the species integrity diagram (Figure 2H). *C. botulinum* can actually be seen as four distinct species that all are able to produce BoNT (Hill et al., 2007; Skarin and Segerman, 2011; Skarin et al., 2011). Some strains without a functional *bont* gene can, from a genomic point of view, be considered to be the same species as *C. botulinum* but they go under other names (e.g., *C. sporogenes, C. novyi).* Furthermore, the *bont* gene can also be found in some *C. baratii* strains and some *C. butyricum* strains.

*Bacillus anthracis* is an example of a species that represents a monophyletic clade within a larger group of related strains (Figure 2I). *B. anthracis* strains all have two virulence plasmids. Apart from the plasmids, they are extremely similar to other related strains (Kolsto et al., 2009). There is a very diffuse boarder between the species in a large group of *Bacillus* strains containing *B. anthracis*, *B. cereus*, *B. thuringiensis*, *B. mycoides, B. weihenstephanensis*, and *B. pseudomycoides*. This group is commonly called the *B. cereus* group (Rasko et al., 2005). Many *B. cereus* strains are much more closely related to *B. thuringiensis* strains than to other *B. cereus* strains.

Finally *Burkholderia pseudomallei* was analyzed (Figure 2J). The most striking observation is its relation to *B. mallei. B. mallei* is a lineage of *B. pseudomallei*, that has undergone massive reductive evolution (Losada et al., 2010). Hence, the pronounced drop in the RCGS values without a corresponding drop in ACGS values.

## Conclusions

When comparing different bacterial species from a genomic perspective, large differences can be found in what we call a species. A large part of the sequencing efforts today are focused on important human pathogens. The niches these species colonize are quite different from those for most environmental species. During their speciation, pathogens have generally shifted from a sympatric to an allopatric lifestyle and this is usually accompanied by reductive evolution and reduced pan genome size. Most of these pathogenic species are more or less distinct, based on core genome conservation, although lateral gene movement channels probably are quite common. In environmental species, however, we see a much larger variability and more poorly defined borders between the species. However, more data are needed on environmental species.

One extreme species is *B. anthracis*. It resides as a spore, without growth. When it does grow, it does so mainly without contact with other bacterial species before returning to the dormant spore state. Thus, it has little opportunity for contact with other bacterial species and hence low genetic variability. On the other extreme is *E coli*. This species has an extraordinarily large and variable set of accessory genes. It also lives in an environment where it is surrounded by a community of different, competing bacteria. Recently, we saw an example of an *E. coli* strain that took up new genetic material by lateral transfer and thereby transformed in to a new, highly virulent variant (Mellmann et al., 2011; Rasko et al., 2011; Rohde et al., 2011).

Other species are located in between these extremes. They may be very close to neighboring species, but are typically still more or less distinguishable based on core sequence similarity. They have specialized in separate, environmental niches. As more strains are sequenced, we will probably find more intermediate forms of these species, showing that the bacterial species concept does not have an absolute definition. However, some draft genomes are of poor quality or are incorrectly annotated and must be treated with caution in any analysis of them.

How should we then relate to bacterial species? For most purposes, species of importance for humans are definable based on the core genome similarity. This is basically what we measure with the classical 16S analysis. However, we must be aware that the species borders are often (perhaps almost always) not absolute, and there will be intermediate strains occurring every now and then. Finally, it is definitely going to be equally important to classify strains according to their accessory gene content. This type of analysis will become much more efficient with the new upcoming sequencing technologies. We are standing at the beginning of a period with an enormous genome database growth and the possibility to greatly increase our understanding of what defines a bacterial species as such.

### Conflict of interest statement

The author declares that the research was conducted in the absence of any commercial or financial relationships that could be construed as a potential conflict of interest.
